# The relationship between family function and the incidence of overweight/obesity in children and adolescents in Chengdu city, Sichuan province of China: based on latent profile analysis

**DOI:** 10.1186/s12889-023-17143-z

**Published:** 2023-11-17

**Authors:** Xixi Jiang, Xiufang Zhao, Junxia Zhou, Xiujuan Zhang, Yan Song, Li Zhao

**Affiliations:** 1 Department of Nursing, West China Second University Hospital, Sichuan University/West China School of Nursing, Sichuan University, Chengdu, China; 2 Key Laboratory of Birth Defects and Related Diseases of Women and Children, Ministry of Education, Sichuan University, Sichuan University, Chengdu, China; 3 Department of Health Policy and Management, West China Fourth Hospital, West China School of Public Health, Sichuan University, 610041 Chengdu, Sichuan China; 4 West China Research Centre for Rural Health Development, Sichuan University, Chengdu, China

**Keywords:** Family function, Overweight, Obesity, Chengdu, Latent profile analysis

## Abstract

**Background:**

Overweight/obesity in children and adolescents has become a global health problem, and family function may be associated with its occurrence. Studies exploring the association between family function and overweight/obesity in children and adolescents were performed in Western and Taiwan, China. To date, related studies haven’t been conducted in Mainland China.

**Objectives:**

To investigate the current status of overweight, obesity, and family function among children and adolescents in Chengdu, China, and to explore their associations.

**Methods:**

Children and adolescents in five primary and middle schools were chosen by cluster sampling. Body Mass Index was used to measure the status of overweight and obesity, and the Chinese family assessment instrument was adopted to assess family function. Latent profile analysis and stepwise logistic regression were applied to identify family classification and explore the relationships between family function and overweight/obesity.

**Results:**

A total of 7616 (84.92%) children and adolescents out of 8968 completed the study with qualified-filled questionnaires. Nine hundred and sixty-six (12.68%)participants were overweight and 656 (8.61%) were obese. The family function was categorized into three profiles: mild (63.93%), moderate (12.32%), and severe (23.75%) dysfunction. The prevalence of overweight was 12.16%, 14.71%, and 13.05% for mild, moderate, and severe family dysfunction, respectively. And the prevalence of obesity was 8.19%, 10.77%, and 8.62% respectively. Participants in moderate and severe dysfunction families were more likely to be overweight (moderate: OR = 1.27, 95% CI:1.01 ~ 1.59, *P* = 0.04; severe: OR = 1.38, 95% CI:1.15 ~ 1.66, *P* = 0.001) and obese (moderate: OR = 1.35, 95% CI:1.02 ~ 1.79, *P* = 0.03; severe: OR = 1.55, 95% CI:1.23 ~ 1.96, *P* < 0.001). Sociodemographic data such as gender, residence, grade, pocket money per week, the number of siblings, and the education level of the mother were all associated with the risk of being overweight/obese in children and adolescents.

**Conclusions:**

The problems of being overweight or obese exist among children and adolescents in Chengdu. And the risk of being overweight or obese increases along with the decrease in family function.

**Supplementary Information:**

The online version contains supplementary material available at 10.1186/s12889-023-17143-z.

## Introduction

Overweight/obesity is defined as abnormal or extreme fat accumulation that may impair health. There are more than 340 million children and adolescents with overweight/obese around the world [1]. China has the most children and adolescents with obesity, and the prevalence of overweight/obesity was expected to increase to 28.0% in 2030, with the absolute number of 49.48 million [2, 3]. Former studies indicated that being overweight/obese during childhood or adolescence had a negative influence on their physical and psychological health, and the impact may continue to their adulthood, causing long-term damage [4–8].

Many factors, such as the consumption of sugar-sweetened beverages [9, 10], screen media use [11, 12], decreasing physical activity [13–15], and inadequate sleep [16] contributed to the occurrence of overweight or obesity. And literature demonstrated that family function might play a role [17–20]. For example, Halliday et al [19]. concluded that more obese individuals were from dysfunctional families, and the specific aspects of family function, like poor communication, high levels of family conflict were linked to an elevated risk of child and adolescent obesity. Other studies [18, 20] also revealed reduction in the risk of obesity in healthy function family. Moreover, investigation [17] conducted in Chinese populations residing in Taiwan and the United States found that family function, rather than dietary behaviors, or physical activities, was associated with overweight/obesity, further emphasizing its importance. Building upon these existing findings, we believed that family function was associated with the risk of being overweight/obese in Mainland China, and sought to explore the associations between them.

Family function emphasizes the interactions among all family members, and how those interactions influence the relationship and functioning of the family as a whole [21]. Some elements, such as connectedness, mutuality, involvement, role assignment, and communication, made up the core of family function, although a series of theories supported their connotations in the specific context [22, 23]. In China, scholars found that emotional expression and communication were less likely to be regarded as attributes of family function, the absence of conflict, interpersonal harmony, mutuality, connectedness, and positive parent-adolescent relationship were considered important compared with the perception of healthy family function in the western context [24]. The issue of children and adolescents overweight/obesity is closely related to family function, as optimal family function promotes individual well-being and development. However, existing studies examining the relationship between children and adolescents’ overweight/obesity and family function have primarily focused on Western countries or Taiwan, China, leaving a research gap in the context of Chengdu, one of the cities in Mainland China. Thus, the objectives of our study were: (1) to investigate the current status of overweight, obesity, and family function among children and adolescents in Chengdu, and (2) to explore the associations between family function and overweight/obesity in this population.

## Method

### Study design

The study employed a cross-sectional design, utilizing data from the baseline investigation of the Chengdu Positive Child Development (CPCD) program. The research was carried out between 23 and 2019 and 13 January 2020 in Chengdu, the capital city of Sichuan province, Mainland China. Before data collection, students were provided with an explanation of the fundamental principles of the collection and utilization of data, including the study’s purpose, voluntary participation, and strict confidentiality measures. The data was collected in the classroom setting, with the presence of two trained research assistants. Each participant independently completed the questionnaires and returned them to the researchers promptly after completing them. To ensure the completeness of the responses, the research assistants and school teachers swiftly reviewed the questionnaires and asked for a refill if necessary. Detailed study designs and methods have been published elsewhere [25].

### Participants

The entire city was categorized into different regions based on economic status, and one district was randomly selected from each region. Subsequently, a school within the chosen district was also randomly selected. To get as much as possible information about the participants, we included all children and adolescents willing to participate in the research from grade 1 to grade 9 and didn’t set specific inclusion and exclusion criteria. A total of 8968 children and adolescents aged 6 ~ 16 in five primary and junior high schools were recruited by cluster sampling.

### Measurement

#### Demographics

The self-designed questionnaire was utilized to gather participants’ demographic information, encompassing factors such as gender, age, ethnicity, residence, number of siblings, grade, weekly pocket money, as well as the education level of both father and mother.

#### Body mass index **(BMI)**

Body mass index (BMI) is a simple and effective index for measuring overweight and obesity. Considering the Chinese context, our research referred to the standard of Chinese school-age children and adolescents released by the National Health and Family Planning Commission in 2018 (see Supplementary 1) [26].

The measurement of children’s height and weight was performed by medical staff from community hospitals. All investigators and medical staff involved in the study underwent training before entering the schools. Height measurements were taken using a stadiometer, with accuracy to the nearest 0.1 cm. Weight measurements were obtained using a digital scale, with accuracy to the nearest 0.1 kg. Boys and girls were instructed to stand upright without shoes or hats, and measured separately.

#### The Chinese family assessment instrument (C-FAI)

The Chinese family assessment instrument (C-FAI) was used to assess family function. C-FAI contains 5 dimensions of family function, including mutuality, communication and cohesiveness, conflict and harmony, parental concern, and parent control. Each dimension includes 3 ~ 12 items with 5 answers (1 = most similar, 5 = most dissimilar), the average score in each dimension was calculated, and a higher score represents the worse performance in the family function dimension [27]. The Cronbach’s alpha was 0.88 in our study, and previous research indicated that C-FAI has good psychometric properties and is an ideal tool to assess the Chinese family function [28, 29].

#### Quality control

Five percent of the questionnaires were randomly selected to verify the data quality: if the selected questionnaires’ consistent rate of entry was equal to or higher than 95%, the data cleaning would be conducted, otherwise, the questionnaires were returned to the participants until the consistent rate reached 95%.

### Data analysis

The data analysis was carried out using SPSS 26.0 and MPlus 7.4 software. We stored all data in SPSS 26.0, removed the missing values and examined for their normal distributions. Descriptive statistics, including measures of central tendency and dispersion, were used to characterize the sample. The division of participants into overweight and obesity categories was accomplished using predefined SPSS syntax [30]. The Mann-Whitney U test and Kruskal-Wallis H were employed to examine the differences in overweight/obesity with different characteristics. Bivariate associations were assessed using Spearman’s correlation analysis. Stepwise logistic regression was employed to investigate the relationship between family function and the occurrence of overweight and obesity based on the results from LPA.

### Latent profile analysis (LPA)

LPA is an exploratory analysis characterized by a person-centered that classifies participants into specific profiles based on their combinations of strengths and weaknesses [31–33]. Before carrying on LPA, researchers always have no information about the profiles of latent variables and need to probe the number of profiles based on the data. All authors of the present literature didn’t know the number of profiles about family function, and it was suitable to apply LPA to identify classifications of families based on individuals’ responses to family function.

First, the one-profile model was adopted in LPA, then profiles were added until the fit indices demonstrated the unfit model. The fit indices of the final profiles models were as follows: Akaike information criterion (AIC) [34], Bayesian information criterion (BIC) [35], sample size-adjusted Bayesian information criterion (aBIC) [36], entropy, adjusted Lo-Mendel-Rubin likelihood ratio test (aLMRT) [37] and bootstrap likelihood ratio test (BLRT) [38]. For AIC, BIC, and aBIC, lower values indicated a better-fitting model. The aLMRT and BLRT statistical significance indicated that the model with the higher number of profiles was better than that with the lower number of profiles [39]. The entropy value evaluated the classification accuracy and ranged from 0 to 1, and a higher value represents the more accurate classification. And 0.8 entropy value is great, representing that more than 90% of families were classified into profiles accurately [40]. The smallest proportion of profile cut-off was 0.05 [41, 42].

## Results

### Participants

A total of 8968 children and adolescents in grades 1 ~ 9 (aged from 6 to 16 years old) participated in the research, and 8825 questionnaires were retrieved. And the qualified questionnaires were 7616 (84.92%). The detailed characteristics of participants were presented in Table 1.

**Table 1:** Demographic information of children (N = 7616)

	N (%) or mean ± SD
Gender (male)	
Boys	3908 (51.31)
Girls	3708 (48.69)
Age (years)	10.65 ± 2.15
Ethnicity	
The Hans	7551 (99.15)
Minorities	65 (0.85)
Residence	
Urban	4876 (64.02)
Rural	2740 (35.98)
The number of siblings	
0	6132 (80.51)
1–2	1403 (18.42)
≥ 3	81 (1.06)
Grade	
Primary school	5187 (68.11)
Junior high school	2429 (31.89)
Pocket money per week (Yuan)	
≤ 20	5599 (73.52)
21–40	982 (12.89)
41–60	651 (8.55)
≥ 61	384 (5.04)
Father education level	
Primary school or below	562 (7.38)
Junior high school	3150 (41.36)
Senior high school	1926 (25.29)
Vocational or technical school	755 (9.91)
College or higher	1223 (16.06)
Mother education level	
Primary school or below	858 (11.27)
Middle school	3037 (39.88)
High school	1788 (23.48)
Vocational or technical school	797 (10.46)
College or higher	1136 (14.92)

### Overweight and obesity

The prevalence of overweight/obesity among our participants was 12.68% and 8.61%, respectively. There were 572 (14.64%) boys and 394 (10.63%) girls who were overweight, while 384 (9.83%) boys and 272 (7.34%) girls were obese. The occurrences of overweight and obesity were 8.26% and 5.93% among participants living in urban areas, compared with that of 4.12% and 2.68% in rural areas. The Mann-Whitney U test suggested boys (Z=-6.11, *P* < 0.001) and participants living in urban areas (Z=-4.19, *P* < 0.001) had higher risks of being overweight/obese. The detailed outcomes were presented in Table 2.

**Table 2:** The prevalence of children and adolescents overweight / obesity

		Non-overweight	Overweight	Obesity	Total	*Z*	*P*	
Gender	Boys	2952 (75.54)	572 (14.64)	384 (9.83)	3908 (51.31)	-6.11	< 0.001	
	Girls	3042 (82.04)	394 (10.63)	272 (7.34)	3708 (48.69)			
Residence	Urban	3772 (49.53)	652 (8.26)	452 (5.93)	4876 (64.02)	-4.19	< 0.001	
	Rural	2222 (29.18)	314 (4.12)	204 (2.68)	2740 (35.98)			
Total		5994 (78.70)	966 (12.68)	656 (8.61)	7616			

### Family function

The scores of the five dimensions in family function were presented in Table 3, and these data were normally distributed with the skewness value ranged from 0.62 to 1.28 and the kurtosis ranged from − 0.31 to 1.37.

**Table 3:** The score of family function

	Mean ± SD	Skewness	Kurtosis
Mut	1.82 ± 0.89	1.28	1.37
Com	1.94 ± 0.95	1.06	0.63
CH	2.10 ± 0.87	0.62	-0.31
PCC	1.70 ± 0.95	1.19	0.50
PCT	2.22 ± 1.17	0.77	-0.37

Notes: Mut = mutuality, Com = communication and cohesiveness, CH = conflict and harmony, PCC = parental concern, PCT = parent control

### Latent profile analysis

The summary of LPA was displayed in Table 4. Three profiles model were selected with indices of entropy (0.89), aLMRT (*P* < 0.001), BLRT (*P* < 0.001) and the smallest proportion of profile (12.32%). The three profiles were named mild, moderate, and severe family dysfunction, respectively. Examining indices of entropy values, BLRT, and aLMRT, all models appeared to be acceptable. The consideration of the smallest proportion among the profiles led us to conclude that the five-profile model wasn’t the most suitable choice, while AIC, BIC, and aBIC suggested that it might be the best fit. Thus, taking all these indices into account, the three-profile model was chosen as the final model.

**Fig. 1:**
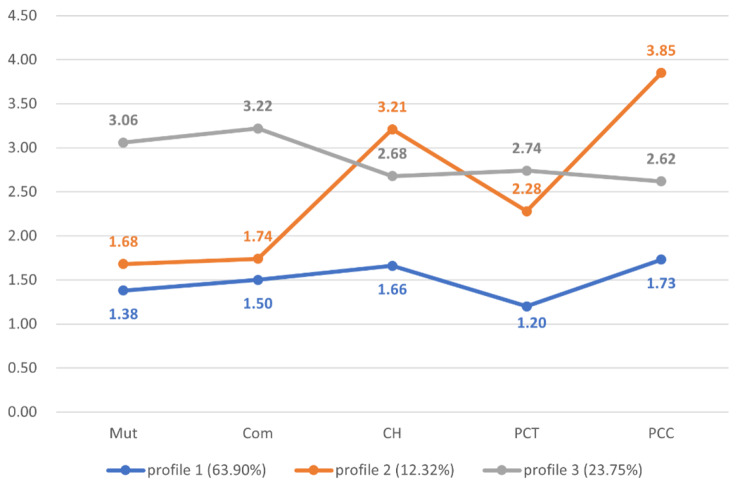
The three-profile model estimated means in each dimension. Notes: Mut = mutuality, Com = communication and cohesiveness, CH = conflict and harmony, PCC = parental concern, PCT = parent control, profile 1 = mild family dysfunction, profile 2 = moderate family dysfunction, profile 3 = severe family dysfunction. Final profile proportions based on the most likely latent profile membership are specified in parenthesis

Figure 1 displayed the estimated means in the 5 dimensions of the three-profile model. The results showed that mild family dysfunction got the lowest mean in each dimension, moderate family dysfunction had the highest means in conflict and harmony and parent control dimensions. Severe family dysfunction possessed the highest scores in mutuality, communication and cohesivenes, and parental concern dimensions.

**Table 4:** The summary of LPA

Model	k	χ2	AIC	BIC	aBIC	entropy	aLMRT	BLRT	Classification probabilities
1	10	-52,414	101,820	104,919	104,887				
2	16	-46,386	92,805	92,916	92,865	0.89	< 0.001	< 0.001	0.7181/0.2819
3	22	-44,302	88,650	88,029	88,732	0.89	< 0.001	< 0.001	0.6390/0.1232/0.2375
4	28	-42,489	85,034	85,228	85,139	0.92	< 0.001	< 0.001	0.5928/0.2773/0.0840/0.0458
5	34	-41,366	82,802	83,038	82,930	0.90	< 0.001	< 0.001	0.5194/0.2016/0.0885/0.1585/0.0320

### Family function, overweight and obesity

Table 5 demonstrated the detailed information about non-overweight, overweight, and obesity in the three types of family dysfunction. The prevalence of overweight/obesity was 12.16%/8.19%, 14.71%/10.77%, and 13.05%/8.62% for families with mild, moderate, and severe dysfunction, respectively. The risk of overweight/obesity was positively associated with increasing levels of family dysfunction (*P* < 0.001).

**Table 5:** Non-overweight, overweight and obesity in three types of family dysfunction

	Non-overweight	Overweight	Obesity	Total(%)
Mild family dysfunction	3878(79.65)	592(12.16)	399(8.19)	4869 (63.93)
Moderate family dysfunction	699(74.52)	138(14.71)	101(10.77)	938 (12.32)
Severe family dysfunction	1417(78.33)	236(13.05)	156(8.62)	1809 (23.75)
Total(%)	5994 (78.70)	966(12.68)	656(8.61)	7616

### Bivariate correlations

Table 6 displayed the result of bivariate correlations, which indicated that gender, ethnicity, the number of siblings, residence, grade, pocket money per week, and the education level of parents might be associated with being overweight/obese, in addition to family function.

**Table 5:** Bivariate correlations

	Gender	Ethnicity	TNOS	Residence	Age	Grade	Edu^1^	Edu^2^	PM	FF	OW/OB
Gender	1.00										
Ethnicity	-0.03	1.00									
TNOS	0.07^**^	-0.02	1.00								
Residence	0.45^**^	-0.04^**^	0.08^**^	1.00							
Age	0.00	0.02	-0.015	-0.21^**^	1.00						
Grade	0.01	0.00	0.031^**^	0.06^**^	0.16^**^	1.00					
Edu^1^	0.00	0.00	0.71^**^	0.07^**^	0.01	0.54^**^	1.00				
Edu^2^	0.03^**^	0.00	0.52^**^	0.04^**^	0.02	0.30^**^	0.63^**^	1.00			
PM	-0.05^**^	0.04^**^	-0.33^**^	-0.07^**^	0.09^**^	0.16^**^	0.07^**^	0.04^**^	1.00		
FF	-0.03^*^	-0.01	0.00	-0.01	-0.25^**^	-0.05^**^	0.00	0.00	0.00	1.00	
OW/OB	-0.08^**^	0.03^*^	-0.10^**^	-0.04^**^	-0.02	0.53^**^	0.39^**^	0.25^**^	0.15^**^	0.02^*^	1.00

Notes: TNOS = the number of siblings; Edu^1^ = father education level; Edu^2^ = mother education level; PM = pocket money per week; FF = family function; OW = overweight; OB = obesity.

* *P*<0.05, ** *P*<0.001

### Stepwise logistic regression

Stepwise logistic regression was applied to further explore the connections between family function and overweight/obesity, family function was regarded as an independent variable, and the other variables were entered as the covariates. Results suggested that the risk of overweight/obesity was higher in the moderate (overweight: OR = 1.27, 95% CI:1.01 ~ 1.59, *P* = 0.04; obesity: OR = 1.35, 95% CI:1.02 ~ 1.79, *P* = 0.03) and severe (overweight: OR = 1.38, 95% CI:1.15 ~ 1.66, *P* = 0.001; obesity: OR = 1.55, 95% CI:1.23 ~ 1.96, *P* < 0.001) dysfunction families when compared with the mild dysfunction family. Gender, residence, grade, pocket money per week, the number of siblings, and mother’s education level might relate to being overweight/obese in children and adolescents. The detailed information was presented in Table 7.

**Table 6:** Stepwise logistic regression outcomes

		*P*	OR	95%CI
Overweight	Mild FD			
	Moderate FD	0.04	1.27	1.01, 1.59
	Severe FD	0.00	1.38	1.15, 1.66
	Gender	0.00	0.65	0.55, 0.77
	Residence	0.02	0.81	0.67, 0.97
	Grade	0.00	8.26	7.03, 9.71
	PM	0.01	1.13	1.04, 1.23
	TNOS	0.00	0.18	0.14, 0.23
	Edu^2^	0.00	1.76	1.62, 1.90
Obesity	Mild FD			
	Moderate FD	0.03	1.35	1.02, 1.79
	Severe FD	0.00	1.55	1.23, 1.96
	Gender	0.00	0.67	0.54, 0.83
	Residence	0.00	0.68	0.55, 0.85
	Grade	0.00	54.89	39.59,76.12
	PM	0.00	1.22	1.10, 1.35
	TNOS	0.00	0.12	0.08, 0.16
	Edu^2^	0.00	1.82	1.65, 2.01

Notes: FD = family dysfunction; PM = pocket money per week; TNOS = the number of siblings; Edu^2^ = mother education level

## Discussion

### Overweight /obesity in children and adolescents

The current research demonstrated that the incidence of children and adolescents overweight was higher than in previous research investigating youngsters from 9 Chinese provinces but not including Chengdu city (12.58% vs. 11.70%) in 2015, while obesity was lower (8.61% vs. 12.74%) [43]. Previous research predicted [3, 44] that the overweight and obesity occurrences were likely to grow sustainably, but our results weren’t following the same tendency, which might have been influenced by the regional disparities. When Comparing the developing and developed countries [45, 46], the prevalence of obesity in Chengdu (boys: 9.83%, girls: 7.34%) was close to the two developing countries like Brazil (boys: 9.2%, girls: 7.6%) and Russian (boys: 9.9%, girls: 7%), but lower than some developed countries like American (boys:16.5%, girls: 15%), Canada (boys:12%, girls: 9.1%), and Korean (boys:17.7%, girls: 12%).

### The risk factors of overweight/obesity

The stepwise logistic model displayed some interesting results. The risk of being overweight in primary school was 8.26 times more than in junior high. For obesity, the OR value even reached 54.89. It’s amazing and suggested that more attention deserves to be paid to junior high school students. Interestingly, only the education level of the mother had a significant association with the youth’s overweight/obesity in the stepwise logistic model, although the bivariate correlation outcomes showed the relationships between both the father’s and mother’s education levels and overweight/obesity. This outcome suggested mothers’ crucial role in being overweight/obese.

LPA categorized family function into three classifications: mild with the lowest scores in all dimensions, moderate with the highest scores in conflict and harmony, and parental control, and severe with the highest scores in mutuality, communication and cohesiveness, and parental concern. Our findings indicated a greater risk of being overweight/obese in families with poorer functioning, consistent with previous research [17, 19]. It is worth noting that Kinston et al [47]. reported an inverse conclusion, associating obesity with better family function. Chen et al [48]. didn’t find significant difference in family functioning among underweight, normal-weight, and overweight children. However, it is well-documented that children from poorly functioning families experience negative health effects and consume fewer servings of fruits and vegetables compared to children from healthy family environments [49–51]. Besides, children growing up in families with healthy functioning are encouraged to engage in physical activities and participate in obesity prevention programs [49, 52]. In a word, more evidence seems to support our conclusion about family function and overweight/obesity.

Family conflict was considered as an environmental stressor, contributing to poor social support in the family [53]. The stress-related reaction was accompanied by increases in corticosteroids and catecholamines, affecting fat storage and blood sugar levels [54]. Moreover, stress has been found to increase the desire to consume highly palatable foods [55]. Therefore, members in a situation with a high level of family conflict for a long time were more likely to develop overweight/obesity [56, 57].

Parental control is a parenting strategy aiming at expressing and maintaining behavioral standards for children. Different levels of parental control could have different effects [24]. Exerting control over food choices and behaviors might prevent overweight/obesity to some extent, particularly for younger children whose food intake (food choice, frequency) is largely dependent on parental decisions. For example, parental control in feeding patterns, such as pressure to eat (like forcing children to eat certain foods), was positively associated with overweight/obesity, but restriction (constraint of child’s access to or intake of certain foods) was not [58, 59]. And early weight status tended to persist throughout a child’s growth. Furthermore, excessive parental control has been demonstrated to be associated with lower fruit and vegetable intake and increased consumption of unhealthy snacks, causing higher BMI and promoting the occurrence of overweight/obesity [60].

Mutuality, Communication, and Parental Concern represent dimensions that are closely related to cohesiveness and communication within the family [28]. Family cohesion refers to the emotional bond shared among all family members [23]. Our findings, along with the research conducted by McConley et al [61]. supported that a cohesive family environment could decrease the risk of overweight/obesity. However, it is worth noting that Hooper et al. [62] suggested that cohesion wasn’t significantly associated with children’s BMI. Nonetheless, the conclusions drawn in their study may be limited by the small sample size.

### Strengths and limitations

The obvious strength of the current research was its sampling method. To represent the situation in Chengdu, all schools were selected from the south, north, west, east, and downtown of Chengdu, and contained enough participants, supplying ample information on family function, the prevalence of overweight/obesity in children and adolescents, and both connections. Another advantage was the application of LPA. LPA divided the family function into three profiles, providing another perspective to identify and further learn the family function specialty.

However, there were certain limitations to consider in the current research. Firstly, the study focused on family function and its associations with overweight/obesity in children and adolescents, but there were multiple factors influencing overweight/obesity. The subsequent follow-up was expected to further identify their connections. The other limitation was the generalizability of our findings beyond Chengdu. The study provided valuable insights into the specific context of Chengdu, caution should be exercised when applying these findings to other regions. Additional research in different settings is necessary to validate and expand upon the conclusions drawn in this study.

## Conclusion

The prevalence of overweight/obesity among children and adolescents in Chengdu was a concern. In this study, the classification of family function into three profiles - mild, moderate, and severe family dysfunction based on individual scores in each dimension, revealed a corresponding increase in the risk of overweight/obesity as the level of family dysfunction escalated. Further research was expected to gain a deeper understanding of the relationships between family dysfunction and overweight/obesity, as well as to explore the generalizability of these findings.

### Electronic supplementary material

Below is the link to the electronic supplementary material.

Supplementary Material 1

## Data Availability

The datasets generated and/or analysed during the current study are available reasonable request to the corresponding author.

## References

[1] Obesity. and overweight. https://www.who.int/news-room/fact-sheets/detail/obesity-and-overweight. Accessed 30 May 2022.

[2] Di Cesare M, Sorić M, Bovet P, Miranda JJ, Bhutta Z, Stevens GA (2019). The epidemiological burden of obesity in childhood: a worldwide epidemic requiring urgent action. BMC Med.

[3] Ma G. Report on Childhood obesity in China. People’s Medical Publishing House; 2017.

[4] Je L, Ht B, Mj H, Jj TW, Th L. F, Being overweight or obese and the development of Asthma. Pediatrics. 2018;142. 10.1542/peds.2018-2119 30478238

[5] Brady TM (2017). Obesity-related Hypertension in children. Front Pediatr.

[6] Quek Y-H, Tam WWS, Zhang MWB, Ho RCM (2017). Exploring the association between childhood and adolescent obesity and depression: a meta-analysis. Obes Rev.

[7] Pulgaron ER, Delamater AM (2014). Obesity and type 2 Diabetes in children: epidemiology and treatment. Curr Diab Rep.

[8] Simmonds M, Llewellyn A, Owen CG, Woolacott N (2016). Predicting adult obesity from childhood obesity: a systematic review and meta-analysis. Obes Rev.

[9] Vs M, Wc AP, Fb W. H. Sugar-sweetened beverages and weight gain in children and adults: a systematic review and meta-analysis. Am J Clin Nutr. 2013;98. 10.3945/ajcn.113.058362 PMC3778861 23966427

[10] Md D, Rj S, Rt D. Sugar-sweetened beverages and weight gain in 2- to 5-year-old children. Pediatrics. 2013;132. 10.1542/peds.2013-0570 PMC3876761 23918897

[11] Mgb JP, So R, Sr S, Lg M, Jc O. B, Effects of limiting recreational screen media use on physical activity and sleep in families with children: a Cluster Randomized Clinical Trial. JAMA Pediatr. 2022;176. 10.1001/jamapediatrics.2022.1519 PMC9127712 35604678

[12] W G, Se J, Rj EMRPIR. B. Association of Video Game Use with Body Mass Index and Other Energy-Balance behaviors in Children. JAMA Pediatr. 2020;174. 10.1001/jamapediatrics.2020.0202 PMC7136857 32250384

[13] Sj M, Sj B. T G, N C, I M. Relationships between media use, body fatness and physical activity in children and youth: a meta-analysis. Int J Obes Relat Metabolic Disorders: J Int Association Study Obes. 2004;28. 10.1038/sj.ijo.0802706 15314635

[14] Drake KM, Beach ML, Longacre MR, Mackenzie T, Titus LJ, Rundle AG (2012). Influence of sports, physical education, and active commuting to school on adolescent weight status. Pediatrics.

[15] Menschik D, Ahmed S, Alexander MH, Blum RW (2008). Adolescent physical activities as predictors of young adult weight. Arch Pediatr Adolesc Med.

[16] F W. H L, Y W, J L, Y C, J Z, Sleep duration and Overweight/Obesity in preschool-aged children: a prospective study of up to 48,922 children of the Jiaxing Birth Cohort. Sleep. 2016;39. 10.5665/sleep.6234 PMC5070755 27568808

[17] Chen J-L, Kennedy C, Family Functioning (2016). Parenting style, and Chinese children’s Weight Status. J Fam Nurs.

[18] Cyril S, Halliday J, Green J, Renzaho AMN (2016). Relationship between body mass index and family functioning, family communication, family type and parenting style among African migrant parents and children in Victoria, Australia: a parent-child dyad study. BMC Public Health.

[19] Halliday JA, Palma CL, Mellor D, Green J, Renzaho AMN (2014). The relationship between family functioning and child and adolescent overweight and obesity: a systematic review. Int J Obes (Lond).

[20] Chen M, Yin W, Sung-Chan P, Wang Z, Shi J (2022). The interactive role of family functioning among BMI status, physical activity, and high-fat food in adolescents: evidence from Shanghai, China. Nutrients.

[21] Minuchin S, Rosman BL, Baker L (1978). Psychosomatic families: Anorexia Nervosa in context.

[22] Dai L, Wang L (2015). Review of Family Functioning. Open J Social Sci.

[23] Normal family processes (2012). Growing diversity and complexity.

[24] Shek DTL, Chan LK. Perceptions of the happy family in a Chinese context. J Youth Stud. 1998;:178–89.

[25] Zhao L, Shek DTL, Zou K, Lei Y, Jia P. Cohort Profile: Chengdu Positive Child Development (CPCD) survey. Int J Epidemiol. 2021;:dyab237. 10.1093/ije/dyab237 35020880

[26] Screening for overweight and obesity among school-age children. and adolescents. http://www.nhc.gov.cn/wjw/pqt/201803/a7962d1ac01647b9837110bfd2d69b26.shtml. Accessed 27 Jun 2022.

[27] Shek DTL (2002). Assessment of Family Functioning in Chinese adolescents: the Chinese Version of the Family Assessment device. Res Social Work Pract.

[28] Siu AMH, Shek DTL (2005). Psychometric properties of the Chinese Family Assessment Instrument in Chinese adolescents in Hong Kong. Adolescence.

[29] Shek DTL, Ma CMS (2010). The Chinese Family Assessment Instrument (C-FAI): hierarchical confirmatory factor analyses and Factorial Invariance. Res Social Work Pract.

[30] Dang J, Yan X, Ma N, Liu Y, Zhong P, Zhang J (2022). Methods for evaluating overweight and obesity among children and adolescents and application in SPSS and SAS. Chin J Prev Med.

[31] Henry NW. Latent structure analysis. Wiley StatsRef: statistics Reference Online. John Wiley & Sons, Ltd; 2014.

[32] Watkins MW, Canivez GL. Are there cognitive profiles unique to students with learning disabilities? A latent Profile analysis of Wechsler Intelligence Scale for Children-Fourth Edition scores. Sch Psychol Rev. 10.1080/2372966X.2021.1919923.

[33] Wang M. Latent variable modleing using Mplus. Chongqing University Press; 2018.

[34] Akaike H, Parzen E, Tanabe K, Kitagawa G (1998). Factor analysis and AIC. Selected papers of Hirotugu Akaike.

[35] Schwarz G (1978). Estimating the dimension of a model. The Annals of Statistics.

[36] Sclove SL (1987). Application of model-selection criteria to some problems in multivariate analysis. Psychometrika.

[37] Birch LL, Davison KK (2001). Family environmental factors influencing the developing behavioral controls of food intake and childhood overweight. Pediatr Clin North Am.

[38] McLachlan D. Finite Mixture models. A Wiley-Interscience Publication; 2000.

[39] Nylund KL, Asparouhov T, Muthén BO (2007). Deciding on the number of classes in latent class analysis and growth mixture modeling: a Monte Carlo Simulation Study. Struct Equation Modeling: Multidisciplinary J.

[40] Lubke G, Muthén BO (2007). Performance of factor mixture models as a function of model size, Covariate effects, and class-specific parameters. Struct Equation Modeling: Multidisciplinary J.

[41] Park K-H, Song MK (2022). Distress among Korean Cancer survivors: a latent Profile Analysis. Int J Environ Res Public Health.

[42] Nasserinejad K, van Rosmalen J, de Kort W, Lesaffre E (2017). Comparison of Criteria for choosing the number of classes in bayesian Finite mixture models. PLoS ONE.

[43] Ma S, Zhang Y, Yang L, Zhao M, Xi B. Analysis on the trend of overweight and obesity of children and adolescents in 9 provinces of China from 1991 to 2015. Chin J Prev Med. 2020;:133-134-135-136-137-8. 10.3760/cma.j.issn.0253-9624.2020.02.004 32074698

[44] Kelly T, Yang W, Chen C-S, Reynolds K, He J (2008). Global burden of obesity in 2005 and projections to 2030. Int J Obes.

[45] World Obesity Federation Global Obesity Observatory. World Obesity Federation Global Obesity Observatory. https://data.worldobesity.org/?_ga=2.145161460.1552752814.1685363386-568977131.1685363386#CN|1|A|F. Accessed 29 May 2023.

[46] Data tables. World Obesity Federation Global Obesity Observatory. https://data.worldobesity.org/tables/prevalence-of-child-overweight-including-obesity-3/. Accessed 2 Jun 2023.

[47] Kinston W, Miller L, Loader P, Wolff OH (1990). Revealing sex differences in childhood obesity by using a family systems approach. Family Syst Med.

[48] Chen J-L, Kennedy C, Yeh C-H, Kools S (2005). Risk factors for childhood obesity in elementary school-age Taiwanese children. Prog Cardiovasc Nurs.

[49] Jerica M, Berge M, Wall N, Larson KA, Loth (2013). Dianne Neumark-Sztainer,. Family Functioning: associations with Weight Status, eating behaviors, and physical activity in adolescents. J Adolesc Health.

[50] Haines J, Rifas-Shiman SL, Horton NJ, Kleinman K, Bauer KW, Davison KK (2016). Family functioning and quality of parent-adolescent relationship: cross-sectional associations with adolescent weight-related behaviors and weight status. Int J Behav Nutr Phys Act.

[51] Alsharairi NA, Somerset SM (2015). Associations between Parenting styles and children’s Fruit and Vegetable Intake. Ecol Food Nutr.

[52] Schmied EA, Madanat H, Chuang E, Moody J, Ibarra L, Cervantes G (2023). Factors predicting parent engagement in a family-based childhood obesity prevention and control program. BMC Public Health.

[53] Cummings EM, Davies PT (2010). Marital conflict and children: an emotional security perspective.

[54] De Kloet ER, Karst H, Joëls M (2008). Corticosteroid hormones in the central stress response: quick-and-slow. Front Neuroendocr.

[55] Klatzkin RR, Baldassaro A, Rashid S (2019). Physiological responses to acute stress and the drive to eat: the impact of perceived life stress. Appetite.

[56] Dallman MF, Pecoraro N, Akana SF, La Fleur SE, Gomez F, Houshyar H (2003). Chronic stress and obesity: a new view of comfort food. Proc Natl Acad Sci USA.

[57] Dallman MF (2010). Stress-induced obesity and the emotional nervous system. Trends in Endocrinology & Metabolism.

[58] Melis Yavuz H, Selcuk B (2018). Predictors of obesity and overweight in preschoolers: the role of parenting styles and feeding practices. Appetite.

[59] Cross MB, Hallett AM, Ledoux TA, O’Connor DP, Hughes SO (2014). Effects of children’s self-regulation of eating on parental feeding practices and child weight. Appetite.

[60] Kiefner-Burmeister A, Hinman N (2020). The role of General Parenting Style in Child Diet and obesity risk. Curr Nutr Rep.

[61] McConley RL, Mrug S, Gilliland MJ, Lowry R, Elliott MN, Schuster MA (2011). Mediators of maternal depression and family structure on child BMI: parenting quality and risk factors for child overweight. Obes (Silver Spring).

[62] Hooper LM, Burnham JJ, Richey R (2009). Select parent and family system correlates of adolescent current weight status: a pilot study. Family J.

